# Baru Proteins: Extraction Methods and Techno-Functional Properties for Sustainable Nutrition and Food Innovation

**DOI:** 10.3390/foods14081286

**Published:** 2025-04-08

**Authors:** Nayara Matiko Reis Miyashita, Eliara Acipreste Hudson, Jaqueline de Paula Rezende, Márcia Cristina Teixeira Ribeiro Vidigal, Ana Clarissa dos Santos Pires

**Affiliations:** 1 Food Technology Department, Federal University of Viçosa, Av. P. H. Rolfs s/n, Viçosa 36570-900, MG, Brazil; nayara.miyashita@ufv.br (N.M.R.M.); eliara.hudson@ufv.br (E.A.H.); marcia.vidigal@ufv.br (M.C.T.R.V.); 2 Food Science Department, Federal University of Lavras, Trevo Rotatório Professor Edmir Sá Santos, s/n, Campus UFLA, Lavras 37203-202, MG, Brazil; jaquelinerezende@ufla.br

**Keywords:** plant-based protein, baru almond, nutritional value, techno-functional property, sustainability, food application

## Abstract

Global population growth raises concerns about the availability of safe and nutritious food, along with its environmental and social impacts. In this context, plant-based foods have emerged as a promising solution, offering sustainable and affordable alternatives. Baru almonds (*Dipteryx alata* Vogel), a native Brazilian species, represent a viable and eco-friendly protein source with significant potential for food applications. This review discusses the nutritional composition, protein extraction methods and techno-functional properties of baru almonds, highlighting both advantages and limitations for food application. Baru proteins exhibit a high protein content (23–30%, *w*/*w*), a balanced essential amino acid profile, and valuable functional properties, including emulsifying capacity, foam stability, and moderate water- and oil-holding capacities. However, despite their potential, the lack of research on the gelation properties of baru proteins restricts their application in structured plant-based food formulations, where protein gelation is crucial for texture, water retention, and overall product stability. Further research is needed to evaluate their gel-forming ability and allergenic potential. Additionally, this review explores emerging protein extraction techniques that could improve protein quality and functionality, expanding their applicability in the food industry. By promoting biodiversity conservation and regional development, baru almonds contribute to the growing demand for sustainable protein sources.

## 1. Introduction

**Figure 1:**
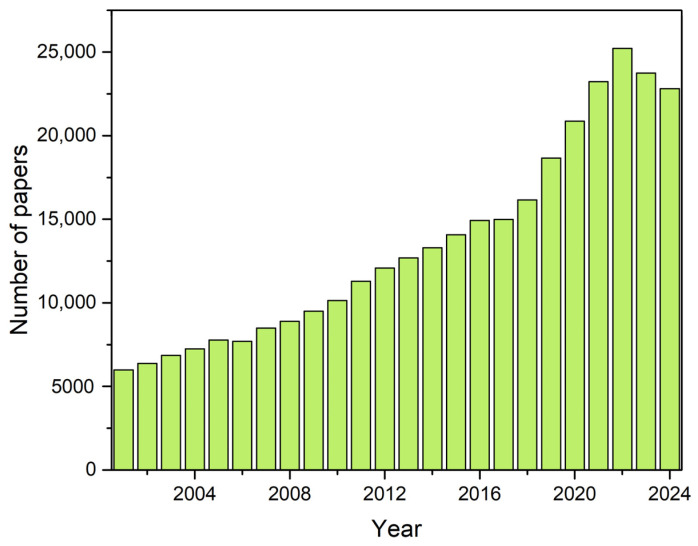
Number of scientific publications reported by Web of Science containing the terms of the topic “plant protein”, “plant-based protein” and “vegetable protein”, from 2001 to 2024.

The demand for plant-based proteins has surged in recent years, driven by population growth and the need for sustainable, nutritious food alternatives that minimize reliance on finite natural resources associated with livestock production [1,2]. Scientific interest in vegetable proteins has increased significantly, with a notable rise in publications from 2001 to 2024 (Figure 1), highlighting their growing relevance across multidisciplinary fields like food science, nutrition, and agronomy.

Many studies have highlighted a prevailing trend toward replacing animal proteins with plant-based proteins, such as soy, wheat, gluten, and pea [3,4,5,6,7]. However, while this shift is evident, it primarily relies on a limited number of conventionally cultivated monoculture crops, which pose significant environmental and social challenges, including soil degradation, biodiversity loss, and the concentration of land and resources [8]. As a result, the long-term sustainability of these protein sources is questionable. The environmental impact of extensive reliance on conventional plant proteins could be comparable to that of meat production. A study assessing the environmental footprint of various burger formulations, incorporating both animal- and plant-based proteins, found that poultry protein had the lowest environmental impact, followed by soybean, pork, lentil, and chickpea protein, based on parameters such as global warming potential, water footprint, energy demand, and ecotoxicity [9].

Furthermore, a lack of dietary diversity may negatively impact health. The global food supply heavily relies on a narrow spectrum of options, with a small number of plant and animal species contributing to 75% of our food intake [10]. This limited food biodiversity can lead to nutritional imbalances, such as unbalanced amino acid profile, reduced protein digestibility, and the excessive presence of antinutritional factors. Moreover, it poses risk of allergic reactions such as those associated with soy, a prevalent component of vegan diets and a recognized allergen by the Food and Drug Administration (FDA) [11]. Generally, plant-based proteins show inferior technological properties compared to animal proteins [12,13], necessitating the blending of multiple proteins in various formulations.

To address these challenges, exploring diverse and sustainable protein sources from global biomes is critical. Unconventional species like baru almonds offer promising nutritional and environmental benefits while supporting regional development and reducing deforestation [14]. Baru almonds reviews have highlighted the health benefits associated with baru consumption [15,16]. However, the specific properties of baru proteins remain relatively unexplored. This review aims to provide a comprehensive understanding of baru almond proteins, particularly concerning their extraction methods, nutritional value, and techno-functional properties for potential use as ingredients in food formulations. Furthermore, the opportunities and challenges involved in incorporating baru proteins into future foods will also be discussed.

## 2. Baru (*Dipteryx alata*): Biome, Characteristics and Almond Composition

The Cerrado, Brazil’s second-largest ecosystem, is among the world’s most biodiverse regions. However, approximately 50% of its area is occupied by agriculture, primarily monoculture, impacting 80% of its native vegetation and risking irreversible degradation by 2030 [17,18]. Sustainable use of native species like baru (*Dipteryx alata* Vogel), offers a solution to preserve biodiversity, reduce deforestation and minimize the environmental impact of long-distance ingredient transport.

**Figure 2:**
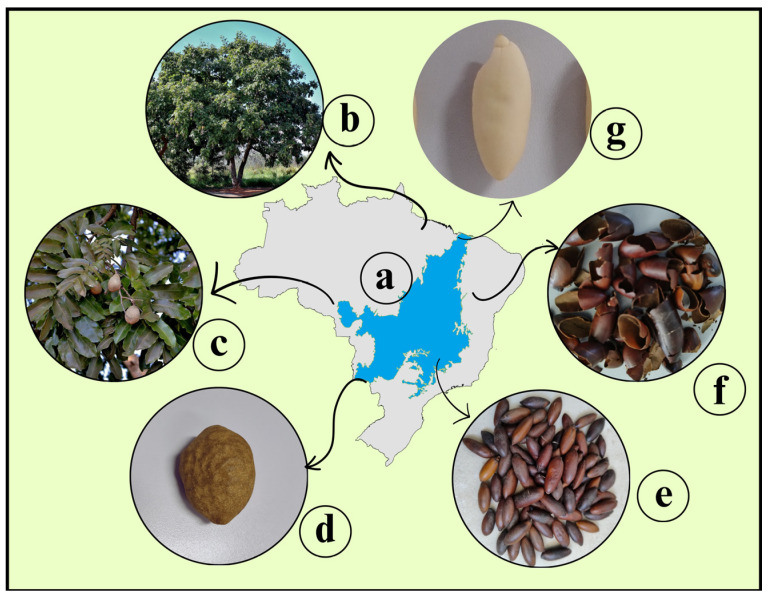
Map of Brazil with the Cerrado region highlighted (a), where baru tree is found (b), baru fruit (c and d), baru almonds with skin (e), its skin (f) and the baru almond without skin (g).

Baru almonds, derived from the fruit of the *Dipteryx alata* tree (Figure 2), are sustainably harvested by rural communities, providing income while minimizing environmental impact. Valued for their nutritional content and peanut-like flavor, baru almonds have a growing global market, projected to rise from USD 5.1 million in 2022 to USD 47 million by 2032 [19].

Nutritionally, baru almonds are rich in protein (23–30%, *w*/*w*), fat (40–45.80%, *w*/*w*), dietary fiber (6.10–12.50%, *w*/*w*), and minerals such as calcium, iron, and zinc [20,21]. Their total protein content exceeds that of almonds (21%, *w*/*w*), pistachio (20%, *w*/*w*), and hazelnut (14.5%, *w*/*w*) [22]. Additionally, raw baru almonds are abundant in bioactive compounds like carotenoids (11.40 µg.100 g^−1^) and tocopherols (11.61 µg.100 g^−1^), contributing to their high antioxidant capacity. However, antinutritional factors like phytates and tannins can reduce nutrient bioavailability. Heat treatment effectively mitigates these compounds, enhancing their nutritional value [23].

Similar to soybean, pea, sunflower, rapeseed, hemp and pumpkin seeds, the predominant protein fraction in baru almonds is globulin (61.7%), followed by albumin, glutelin, and prolamin, highlighting its potential for diverse food applications [22,24]. The application of baru almonds as food ingredients aligns with global efforts to promote underutilized crops for sustainable and nutritious food solutions.

## 3. Nutritional Proprieties of Baru Proteins

The quality of proteins is determined by their essential amino acids (EAAs) content, digestibility, bioavailability, purity, and the impact of extraction and processing methods. Table 1 compares the amino acid profile of baru almond proteins with other high-protein sources. Baru almond flour contains 25.81 g/100 g of protein, lower than soybean flour (41.85 g/100 g), and animal-derived powders like eggs (48.56 g/100 g) and casein (81.57 g/100 g), but higher than other vegetable sources like pea flour (21.50 g/100 g), and *Moringa oleifera* leaves (moringa) (23.60 g/100 g), making it valuable source of protein, particularly in vegetarian and plant-based diets.

The concentration of EAAs in proteins from animal sources such as dehydrated beef, casein powder, and egg powder meets the dietary recommendations of the FAO/WHO/UNU reference pattern [25]. In contrast, plant-based proteins often contain limiting amino acids. For example, soybean protein is deficient in methionine + cysteine (18.65 mg/g), making it necessary to combine it with other protein sources rich in these amino acids. Conversely, *Moringa oleifera* flour contains an excess of methionine + cysteine (71.40 mg/g) but is limited in histidine, isoleucine, and leucine. Given these differences, combining different plant protein sources, such as soybean and *Moringa oleifera* flour, could enhance amino acid balance and improve protein quality in food formulations.

However, baru almond flour demonstrates a robust EAA profile. Baru almond flour contains significant amounts of methionine + cysteine (22.00 mg/g of protein), histidine (23.4 mg/g of protein), isoleucine (37.5 mg/g of protein), and leucine (77.8 mg/g of protein), which indicates a well-balanced composition that aligns closely with the FAO/WHO/UNU dietary recommendations. When compared to the FAO reference values, baru almond flour meets or exceeds all these standards [25,26]. For example, compared to soybean flour—the most widely used plant-based protein in the food industry—baru almond flour presents higher methionine + cysteine (22.08 mg/g vs. 18.65 mg/g) and valine (51.8 mg/g vs. 48.16 mg/g) content. However, it falls short in other EAA, which can be attributed to the higher overall protein content in soybean flour. Despite this, the well-balanced and complete EAA profile of baru almond flour stands out, highlighting its potential as a nutritious alternative protein source.

Regarding pea flour, both baru almond and pea flour do not present limiting amino acids and show similar methionine + cysteine. Nevertheless, baru almond flour is superior in leucine (77.8 mg/g vs. 70.00 mg/g), phenylalanine + tyrosine (87.33 mg/g vs. 65.00 mg/g), threonine (44.90 mg/g vs. 37.00 mg/g), and valine (51.8 mg/g vs. 39.00 mg/g). When compared to another unconventional protein source, *Moringa oleifera* flour, baru almond flour shows no limiting amino acids, whereas *Moringa oleifera* leaves present limiting amounts of histidine (14.40 mg/g), isoleucine (28.80 mg/g), and leucine (56.60 mg/g). However, baru almond flour has significantly lower methionine + cysteine (22.08 mg/g vs. 71.40 mg/g) and lysine (48.4 mg/g vs. 58.20 mg/g) content. Over again, baru almond amino acid profile stands out for being a well-equilibrated source of EAA, as promising as moringa, but it does not present the dark green color and astringent flavor, which can affect sensory properties of foods [32].

Baru almond flour demonstrates high in vitro protein digestibility (IPD: 86.0%), comparable to animal-based proteins and superior to many plant-based options, including soybean and moringa flours. Its digestibility enhances the delivery of essential amino acids, making it suitable for diverse applications, including breads, snacks, vegan supplements, and protein bars. As global demand for plant-based proteins grows, baru almond flour emerges as a sustainable and innovative option that supports nutrition and biodiversity.

## 4. Extraction of Baru Almond Proteins

Baru almond protein extraction plays a crucial role in determining its functional properties, beginning with oil removal, typically performed via mechanical pressing, producing defatted baru almond flour (DBF), a protein-rich byproduct [33,34]. Combining mechanical pressing with solvent extraction improves oil recovery and protein yield while minimizing oxidation [35]. Safer solvents like ethanol and supercritical CO_2_ are preferred over traditional hexane for enhanced safety and efficiency [36]. 

Baru protein concentrate (BPC) and isolate (BPI) are obtained via alkaline extraction. This method is also applied to other plant sources, including soy, almonds, and peanuts. DBF is solubilized in an alkaline medium (pH~10) to enhance protein solubility through electrostatic repulsion, followed by centrifugation to collect the protein-rich supernatant for BPC production. Further acidification of supernatant to the isoelectric point (pI) (pH~4.8), causes protein aggregation, resulting in the BPI [37].

This process yields BPC and BPI with a high protein content (55.03 g/100 g and 84.11–88.4 g/100 g, respectively), comparable to or exceeding other protein isolates sources like soy (87.6 g/100 g) and casein (83.0 g/100 g), highlighting the feasibility and competitiveness of baru protein extraction for use in food formulation [37,38]. Despite its high efficiency, simplicity, and cost-effectiveness, the alkaline extraction-acid precipitation method has some drawbacks. It can lead to protein denaturation and the formation of insoluble aggregates, negatively impacting digestibility and techno-functional properties [39,40,41,42]. Additionally, the alkaline extraction generates large amounts of acid and alkali wastewater as environmental pollutants and may form toxic species, such as lysinoalanine, having a significant environmental footprint [41].

The Osborne method offers a milder alternative that allows the sequential separation of different protein fractions without the formation of toxic species, also, unlike alkali protein extraction, preserves the functionality of albumins and globulins, the main protein fractions in baru almonds. This sequential extraction is based on solubility, using water (pH 7.0, 1:30 *w*/*v*) for albumins, followed by 0.5 mol/L NaCl (pH 7.0, 1:30 *w*/*v*) for globulins, 700 g/L ethanol for prolamins, and 0.1 mol/L NaOH for glutelins. Stirring (60 min) and centrifugation (32,000× *g*, 40 min) were applied at each step, with dialysis (10 kDa membranes, 24–36 h) where applicable. This method recovers 80% of total baru almond proteins, with globulins (61.7%) as the predominant fraction, followed by albumins (14.0%), glutelins (3.3%), and prolamins [22]. Despite its effectiveness, the Osborne method is time-consuming and requires precise handling, limiting its industrial scalability [43].

Innovative techniques such as enzyme-assisted (EA) extraction reduce harsh chemical usage while improve protein yield and techno-functional properties by enhancing emulsification, solubility, and water- and oil-holding capacities, as observed in chickpea protein properties [44,45]. It is particularly suitable for heat-sensitive ingredients, but its commercial application is limited by high enzyme costs, sensitivity, and the potential formation of bitter byproducts [46].

Ultrasound-assisted extraction (US) is a sustainable alternative due to low energy consumption, maintenance costs, and processing time. When combined with conventional techniques, it increased protein yields from Spirulina (32.48% to 76.83%, 200 W, 26 kHz), while enhancing solubility and digestibility [47,48]. Despite its advantages, US protein extraction faces challenges such as heat generation, free radical formation, and limited scalability [49].

Pulsed electric field (PEF) is an eco-friendly and cost-effective protein extraction technique, that enables by-products recovery from food waste. Combining PEF with alkaline extraction (2.3 kV for 25 min) improved rice bran protein yield by 22.8% and enhanced functional properties, including oil-holding capacity and emulsifying (20.29–22.64%, 3.3–12.0%, respectively) [46]. However, high initial equipment costs and process optimization remain challenges for large-scale applications [50].

Deep eutectic solvents (DES), formed from a hydrogen bond acceptor (HBA) and donor (HBD), are non-toxic, recyclable, and cost-effective, making them suitable for protein extraction. For instance, a DES made from choline chloride (HBA) and levulinic acid (HBD), in a 6:1 molar ratio, extracted 39.16 mg/g of protein from bamboo shoots, outperforming conventional methods (23.88 mg/g) [51]. Despite their potential, further research is needed to assess their compatibility, biodegradability, and recyclability for broader food industry applications.

Integrating emerging extraction methods can enhance protein yield and functionality, making baru almonds proteins valuable ingredients for diverse food applications. Nevertheless, selecting an appropriate extraction approach requires balancing efficiency, cost, and sustainability to minimize environmental impact while maximizing protein recovery. Hybrid strategies, combining conventional and green technologies, may offer an optimal compromise for scalable and eco-friendly industrial applications.

## 5. Baru Almonds Techno-Functional Properties

Proteins play a crucial role in food product development, influencing sensory characteristics and techno-functional properties. Baru almond proteins offer a promising plant-based alternative, making essential to evaluate their functionality and competitiveness with other protein sources. The next sections will compare the techno-functional properties of baru almonds with animal and plant proteins.

### 5.1. Solubility

**Figure 3:**
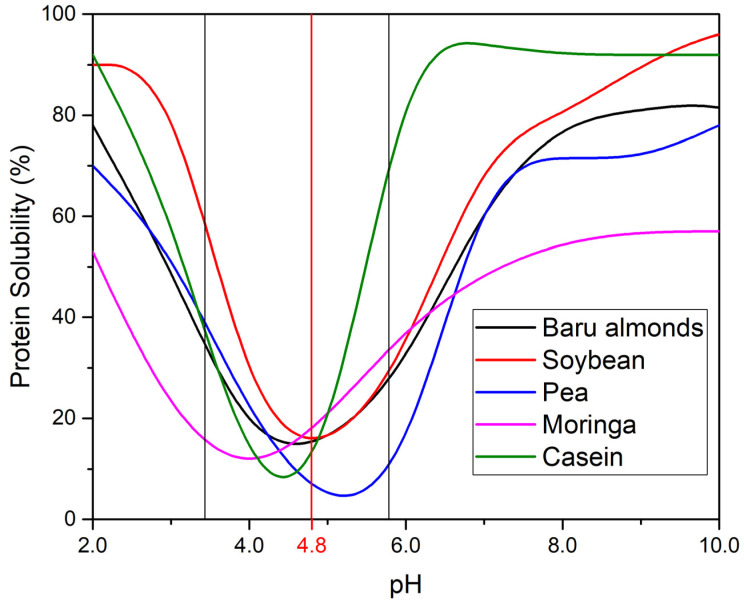
Solubility profile of proteins from baru almond [37], soybean [52], *Moringa oleifera* leaves [53], pea [54] and sodium caseinate [55], with their isoelectric point (pI) region indicated by vertical black lines. The pI of baru almond proteins (pI = 4.8) is specifically marked with a vertical red line. Adapted with permission from [37], 2016, John Wiley and Sons; ref. [52] 1976, American Chemical Society, ref. [54], 2013, Elsevier, ref. [55], 2009, Cambridge University and ref. [53], 2020, Creative Commons license.

Protein solubility is a foundational property influencing applications like emulsification, foaming, and gelation. Baru proteins follow a U-shaped solubility curve relative to pH, similar to other plant-based proteins like soybean, pea, and moringa (Figure 3). At alkaline pH (8–10), baru proteins show high solubility (approximately 83%), surpassing pea (80%), and moringa (58%) proteins but slightly below soy, whey, egg, and sodium caseinate proteins, which exceed 88%. Above their pI, proteins exhibit a net negative charge, increasing mutual repulsion and solubility.

At acidic pH (3–5), near to their pI, baru proteins experience a sharp decrease in solubility, similar to soy protein isolate and sodium caseinate, which form precipitates near their pI. This reduced solubility limits their use in low-pH products like beverages and acidic dressings, where high solubility is essential for stability.

Several strategies have been proposed to improve the solubility of plant proteins in acidic conditions. Enzymatic hydrolysis, which generates peptides with enhanced solubility [56], and complexation with highly soluble proteins, such as whey proteins, have both been shown to enhance protein solubilization and resistance to aggregation [57]. Additionally, modifications such as the use of sodium alginate under microwave radiation [58] and the combination of pulsed electric field with pH shifting have demonstrated improvements in protein solubility [59]. Future research could explore the use of stabilizers, such as gum Arabic [60], in combination with baru protein isolate to improve protein stability under acidic conditions, potentially broadening their application in various food matrices by preventing precipitation and increasing solubility at acidic pH.

Despite their limitations at acidic pH, the solubility profile and mild flavor of baru proteins position them as a promising alternative to soy in neutral pH applications, such as plant-based beverages. With plant proteins representing only 18% of protein-rich beverages, the underrepresentation in this market highlights a significant opportunity for innovative product development using baru proteins [61].

The solubility of baru proteins across different pHs plays a crucial role in their incorporation into aqueous systems and in enhancing their functional properties, such as emulsification. This functionality is essential in stabilizing food systems, including emulsified products like non-dairy creams and protein-enriched drinks, further expanding the potential uses of baru proteins in the food industry.

### 5.2. Emulsifying Activity and Stability

Emulsifying capacity (EC) measures the efficiency of proteins emulsification and it is calculated as the ratio of emulsion layer height to the total mixture height after mechanical emulsification. Emulsifying kinetic stability (ES) evaluates the percentage of emulsion that remains stable over time, determined by the ratio of initial to remaining emulsion volume after stress tests like centrifugation, heating, or storage [62]. Table 2 presents the emulsifying properties of baru proteins, offering a comparison with other high-protein sources.

The DBF showed EC and ES (51 and 50%, respectively) slightly smaller than BPC (55 and 51%, respectively) and BPI (55 and 54%, respectively). Despite the higher protein content of BPI (84.11 g.100 g^−1^) compared to DBF (49.00 g.100 g^−1^) and BPC (57.65 g.100 g^−1^), there is no significant improvement in EC and ES, suggesting that these properties depend more on the quality than the quantity of soluble proteins [37,62,72]. The emulsifying properties of baru almond proteins are largely attributed to their high globulin content, typical of legume seeds like soybeans. Globulins are amphipathic, with both hydrophobic and hydrophilic regions, allowing them to adsorb to the oil–water interface and reduce interfacial tension. This enables them to stabilize oil-in-water emulsions effectively. The molecular structure of globulins, which facilitates their ability to form stable layers around oil droplets, makes them efficient emulsifiers in various food applications [73,74].

Compared to other proteins, BPI offers competitive emulsifying properties. It surpasses sodium caseinate (30%) and *Moringa oleifera* protein isolate (20%) in EC, demonstrating its potential as a robust plant-based emulsifier. Furthermore, BPI’s EC is comparable to that of soy (48.2%) and pea protein isolates (57.1%), both widely used plant-based emulsifiers in foods like cereals, bakery, and meat products. However, BPI EC is lower than whey protein isolate (73.4%), which is known for its superior solubility and interfacial activity. Whey, soy, and egg proteins are often associated with allergenic reactions, making it essential to evaluate the allergenicity of baru proteins to ensure they meet low-allergenicity standards.

At neutral pH, baru proteins display adequate EC and emulsifying stability (ES), making them suitable for plant-based beverages. Defatted baru flour (DBF) has shown potential as a wheat flour substitute, with 25% DBF cookies achieving sensory acceptance comparable to wheat-based counterparts and enhancing nutritional value [75]. Baru almonds have also been utilized in innovative products, such as protein-enriched cereal bars and vegan “Dulce de leche”, both receiving high sensory scores (higher than 7) for appearance, flavor, and texture [14,76].

The EC and ES of baru almond proteins, particularly in the forms of defatted flour, protein concentrate, and isolate, highlight their potential as versatile emulsifying agents in various food applications. Also, baru almond proteins may offer advantages as a lower-allergen alternative to traditional emulsifiers, pending further studies on allergenicity. These findings suggest that baru almond proteins could serve as valuable ingredients in developing plant-based food products and clean-label formulations, expanding their potential applications in the food industry.

### 5.3. Foam Capacity and Stability

Foam capacity (FC) and foam stability (FS) are essential properties in aerated food products, influencing texture and structure in products like breads, cakes, meringues, and ice creams. FC refers to the volume of foam that proteins generate, while FS describes the protein’s ability to stabilize the foam against gravitational and mechanical stress.

FC and FS values for baru proteins and other proteins are presented in Table 2. BPI demonstrates an FC of 58.30%, surpassing soybean protein isolate (SPI, 25.00%) and *Moringa oleifera* protein isolate (13.00%). Conversely, higher SPI FC values reported in other studies (82.00% to 250.00%) highlight the influence of blending speed and time, suggesting similar adjustments could enhance the FC of BPI [64,77,78].

The FC and FS of BPC (95.00% and 23.00%, respectively) and DBF (69.00% and 35.00%, respectively) differ from BPI (58.30% and 96%, respectively), reflecting the impact of extraction processes on protein structure. In BPI, globulins are the main protein fraction, forming viscoelastic films that enhance foam stability [79,80]. Although its FC is lower than whey (320.00%), pea (300.00%), and sodium caseinate (290.00%), its superior FS (96.00% vs. 35–58.00%) makes it ideal for stable foam applications like whipped toppings and vegan meringues.

Future research could explore how various processing techniques influence the foaming properties of plant proteins, potentially enhancing their functionality in food applications. For instance, high-intensity ultrasound has been shown to modify the structure of soy protein isolates, leading to improved foaming capacity by reducing particle size and altering interfacial properties [78]. Similarly, heat treatments can affect the surface hydrophobicity and solubility of soy protein concentrates, thereby enhancing their foaming properties [81]. Additionally, the glycation reaction, when combined with ultrasound treatment, has been reported to improve the foaming properties of brewer’s spent grain protein [82]. Investigating these and other processing methods, such as enzymatic hydrolysis and pH modification, could provide valuable insights into optimizing the foaming characteristics of plant proteins for diverse food applications.

### 5.4. Gelation

Gelation (GE) is a key techno-functional property for developing structured plant-based foods, such as meat, egg, yogurt, and cheese analogs. It typically occurs through thermal denaturation, where protein molecules unfold, aggregate, and form a protein network.

While extensive studies have focused on the emulsifying and foaming capacities of baru proteins, their gelation properties remain largely unexplored, representing a critical knowledge gap. Considering insights from other plant-based proteins, as soy proteins (12%) and pea proteins (14%), they require higher concentrations compared to animal proteins like ovalbumin (1%) to achieve gelation [67,83,84]. These variations highlight how protein type and molecular composition influence gelation behavior. Smaller globular proteins, such as those predominant in pea protein, require higher concentrations to form a gel. Since globulins are the major protein fraction in baru almond, further research is needed to determine the least gelation concentration of its protein concentrates and isolates, as well as the gelation behavior of individual protein fractions. Additionally, studies on their denaturation temperature and its impact on gel strength are essential to better understand their functionality [22,83,85].

Given the unique protein profile of baru almonds, investigating the gelation properties of baru proteins under various conditions may further expand their application in different food matrices, offering functional and textural benefits that align with the growing demand for plant-based, texture-optimized foods. Future research should explore the effects of protein concentration and thermal treatments on the gelation behavior of baru proteins. Studies on almond proteins have shown that heating protein solutions (3.6% protein (*w*/*v*), 85–95 °C, 30 min) can induce gel formation with high water-holding capacity and gel strength comparable to dairy gels [86]. Additionally, enzymatic hydrolysis has been reported to modify the gelation properties of almond proteins (8% protein (*w*/*v*), 85 °C, 30 min), where limited hydrolysis (degree of hydrolysis less than 10%) resulted in gels with improved water-holding capacity and texture [87]. Investigating similar conditions for baru proteins, including varying protein concentrations, thermal treatments, and enzymatic modifications, could provide valuable insights into optimizing their gelation properties for diverse food applications.

### 5.5. Water- and Oil-Holding Capacity

Water-holding capacity (WHC) measures a protein’s ability to retain water. In applications such as soups, baked goods, meat analogues, sausages, and custards, high WHC helps maintain moisture and product stability. For example, in meat analogues, plant proteins with high WHC contribute to replicating the texture and juiciness of traditional meat. In emulsified meat products, such as sausages and mortadella, WHC influences gel formation, ensuring product integrity and preventing phase separation. On the other hand, oil-holding capacity (OHC) indicates a protein’s ability to retain oil, enhancing flavor, mouthfeel, and structure in lipid-rich foods. In cheeses and sauces, high OHC helps maintain a creamy and homogeneous texture. Additionally, OHC plays a crucial role in restructured meats and meat substitutes, where oil retention improves texture and enhances the sensory profile of the final product [88,89,90,91,92].

Interestingly, studies in the literature have shown that the WHC of baru proteins decreases as protein concentration increases, with DBF exhibiting the highest WHC value (225.98%, 49.00 g·100 g^−1^), followed by BPC (193.84%, 57.65 g·100 g^−1^) and BPI (118.30%, 88.40 g·100 g^−1^) [37,62]. This decline in WHC for BPI is likely associated with conformational changes induced during the acid precipitation step during protein extraction, leading to partial protein aggregation and reduced structural flexibility [58]. Additionally, this step further alters protein conformation, decreasing the availability of hydrophilic sites and contributing to WHC reduction [93]. Similarly, OHC shows a decreasing trend, with DBF exhibiting the highest value (199.80%) compared to BPI (155.50%), likely due to the loss of low-molecular-weight protein fractions, such as albumins and globulins, during the extraction process [37,62].

Baru proteins have a distinct amino acid profile that contributes to their functional properties. Hydrophilic amino acids like lysine and glutamine enhance WHC, while hydrophobic residues such as leucine and phenylalanine improve OHC [26,94,95]. Compared to other isolates, BPI’s WHC is lower than pea (300–430%), soybean (310.90%), and sodium caseinate (300%), but higher than whey protein isolate. This balance between moisture retention and solubility supports its use in formulations requiring enhanced texture and stability.

BPI demonstrates a higher OHC (155.50%) than soybean protein isolate (60.00%), enhancing its suitability for fat-rich foods, but it falls short of whey protein isolate (320.00%) and sodium caseinate (280.00%) [37,66,69]. The superior OHC of caseins are attributed to their flexible structures, which expose more hydrophobic sites compared to the globular proteins in baru almonds. These differences highlight BPI’s potential for specific lipid-rich food applications, despite structural limitations.

Furthermore, OHC of BPI (155.50%) is comparable to the lower range of pea (110–250.00%), and *Moringa oleifera* leaves (160–355.00%) protein isolates, likely due to the similar globulin composition of pea and baru proteins. However, the specific proteins in baru almonds remain unidentified, warranting further research to better understand and optimize BPI’s structural properties for targeted industrial applications.

These intermediate WHC and OHC values position baru proteins as a promising alternative for food formulations requiring moderate water and oil retention, such as meat substitutes, sauces, and bakery products. Moreover, the balanced amino acid profile and compatibility with other protein sources highlight their potential as a functional ingredient.

## 6. Conclusions

Baru almond proteins hold significant potential as a sustainable and versatile ingredient for the food industry. Their balanced nutritional profile, which meets all FAO essential amino acid standards and their functional properties make them ideal for incorporation into diverse food products, including vegan and plant-based formulations. Advanced extraction methods can optimize their usability and efficiency, supporting industrial scalability. Furthermore, the utilization of baru almonds promotes Cerrado conservation and regional socio-economic development, aligning with global sustainability goals.

However, despite their promising functional characteristics, the gelation properties of baru proteins remain underexplored. A deeper understanding of their gel-forming behavior is crucial for expanding their applications in food systems, particularly in texturized plant-based products. Continued research on optimizing their functionality, including gelation mechanisms and allergenic potential, is essential to fully harness their applications in innovative food formulations.

## Figures and Tables

**Table 1:** Essential amino acid profile (mg.g^−1^ of protein), total protein content (g.100 g^−1^) and in vitro protein digestibility (%) of high-protein sources.

	FAO [25]	Baru Almond Flour [22,26]	Soybean Flour [27,28]	Dehydrated Beef [28]	Bovine Casein Powder [28]	Eggs Powder [28,29]	*Moringa oleifera* Flour [30] *	Pea Flour [31]
	EAA		Amino acid concentration (mg.g^−1^ of protein)					
Met + Cys	22	22.00	18.65	35.59	30.14	40.05	71.40	23.00
His	15	23.40	32.88	38.10	18.99	22.12	14.40	35.00
Ile	30	37.50	37.36	39.43	46.91	34.64	28.80	35.00
Leu	59	77.80	81.34	92.24	93.05	83.90	56.60	70.00
Lys	45	48.40	82.69	95.28	78.66	91.44	58.20	63.00
Phe + Tyr	38	87.33	96.99	83.86	109.71	98.64	74.10	65.00
Thr	23	44.90	51.34	48.23	43.22	53.50	39.00	37.00
Trp	6.0	20.20	n.d.	n.d.	n.d.	n.d.	17.60	n.d.
Val	39	51.80	48.16	43.00	54.95	47.52	42.80	39.00
		Total protein content (g.100 g^−1^)						
		25.81	41.85	81.76	81.57	48.56	23.60	21.50
IPD (%)		86.00	78.90	80.00	91.00	81.40	57.22–64.70	86.20–86.90

n.d: not determined. Tryptophan could not be analyzed due to the degradation of this amino acid during acid hydrolysis, which was used during sample preparation. IPD: In vitro protein digestibility. * *Moringa oleifera* flour was added as another example of non-conventional protein.

**Table 2:** Techno-functional properties of baru almonds and other high proteins sources.

	Emulsifying Capacity (EC) (%)	Emulsifying Stability (ES) (%)	Foam Capacity (FC) (%)	Foam Stability (FS) (%)	Water- Holding Capacity (%)	Oil-Holding Capacity (%)	Experimental Conditions
Baru almond protein isolate (BPI) [37]	54.80	53.90	58.30	96.0	118.30	155.50	pH 7.0; ES: 70 °C/30 min; FS: 60 min.
Baru almond protein concentrate (BPC) [62]	55.00	51.00	95.00	23.00	193.84	205.28	pH 7.0; ES: 80 °C/30 min; FS: 60 min.
Defatted baru almond flour (DBF) [62]	51.00	50.00	69.00	35.00	225.98	199.80	pH 7.0; ES: 80 °C/30 min; FS: 60 min. after.
Soybean protein Isolate (SPI) [37]	48.20	47.50	25.00	92.00	310.90	60.00	pH 7.0; ES: 80 °C/30 min; FS: 60 min.
*Moringa oleifera* leaf protein isolate [53,63]	20.00	35.00	13.00	4.00	231–280.00	160–355.00	pH 6.0; ES: 70 °C/30 min; FS: 10 min.
Sodium caseinate [64,65,66]	30.00	19.50	290.00	40.00	300.00	280.00	pH 7.0; ES: 80 °C/30 min; FS: 30 min.
Pea protein isolate [54,64,67]	57.10	60.00	300.00	58.00	300–430.00	110–250.00	pH 7.0; ES: 7 days; FS: 5 min.
Whey protein isolate [64,68,69,70,71].	41.00	57.65	320.00	35.00	20.00	320.00	pH 7.5; ES: 10 min; FS: 30 min.

## Data Availability

No new data were created or analyzed in this study.

## Abbreviations

The following abbreviations are used in this manuscript:

BPC	Baru protein concentrate
BPI	Baru protein isolate
DBF	Defatted baru almond flour
DES	Deep eutectic solvents
EA	Enzyme-assisted extraction
EAAs	Essential amino acids
EC	Emulsifying capacity
ES	Emulsifying kinetic stability
FC	Foam capacity
FS	Foam stability
GE	Gelation
HBA	Hydrogen bond acceptor
HBD	Hydrogen bond donor
IPD	In vitro protein digestibility
OHC	Oil-holding capacity
PEF	Pulsed electric field
pI	Isoelectric point
SPI	Soybean protein isolate
US	Ultrasound-assisted extraction
WHC	Water-holding capacity
